# High-speed dynamic-mode atomic force microscopy imaging of polymers: an adaptive multiloop-mode approach

**DOI:** 10.3762/bjnano.8.158

**Published:** 2017-08-02

**Authors:** Juan Ren, Qingze Zou

**Affiliations:** 1 Department of Mechanical Engineering, Iowa State University, 2030 Black Engineering, Ames, IA 50011, USA; 2 Department of Mechanical and Aerospace Engineering, Rutgers University, 98 Brett Rd, Piscataway, NJ 08854, USA

**Keywords:** adaptive multiloop mode, atomic force microscopy (AFM), heterogeneous polymer sample, tapping-mode imaging

## Abstract

Adaptive multiloop-mode (AMLM) imaging to substantially increase (over an order of magnitude) the speed of tapping-mode (TM) imaging is tested and evaluated through imaging three largely different heterogeneous polymer samples in experiments. It has been demonstrated that AMLM imaging, through the combination of a suite of advanced control techniques, is promising to achieve high-speed dynamic-mode atomic force microscopy imaging. The performance, usability, and robustness of the AMLM in various imaging applications, however, is yet to be assessed. In this work, three benchmark polymer samples, including a PS–LDPE sample, an SBS sample, and a Celgard sample, differing in feature size and stiffness of two orders of magnitude, are imaged using the AMLM technique at high-speeds of 25 Hz and 20 Hz, respectively. The comparison of the images obtained to those obtained by using TM imaging at scan rates of 1 Hz and 2 Hz showed that the quality of the 25 Hz and 20 Hz AMLM imaging is at the same level of that of the 1 Hz TM imaging, while the tip–sample interaction force is substantially smaller than that of the 2 Hz TM imaging.

## Introduction

In this paper, the adaptive-multiloop imaging mode of atomic force microscopy (AFM) is tested and evaluated by imaging three largely different heterogeneous polymer samples. AMLM imaging substantially increases the speed of tapping mode (TM) imaging (by over an order of magnitude) while preserving the advantages of TM imaging over contact mode (CM) imaging [1]. Although TM imaging is the de facto most widely used imaging technique of AFM [2–3], the slow speed (throughput) of TM imaging has become its major limit and bottleneck [4–5]. It is challenging to achieve high-speed TM imaging because an increase of the speed can cause a loss of the tip–sample interaction and/or the annihilation of the cantilever tapping vibration, particularly when the imaging size is large. Existing efforts on high-speed TM imaging [6–9] only led to a speed increase up to three times at the cost of a substantially (over five times) increased imaging force. By using the AMLM imaging mode, it is aimed to achieve high-speed dynamic-mode AFM imaging while maintaining the tip–sample interaction force similar as that in low-speed TM imaging.

The speed increase of TM imaging is limited by the control mechanism applied [4,6]. Due to the time delay inevitably induced into the feedback loop for maintaining the RMS tapping amplitude during imaging, errors in tracking the sample topography can quickly result in loss of the tip–sample contact and annihilation of the probe tapping when the imaging speed increases [3,6]. This is because the tapping amplitude is sensitive and highly nonlinear with respect to the tip–sample distance [10–11]. The speed of TM-imaging might be increased through either hardware [12–14] or software (algorithms) improvement [6,8,15–16]. However, the existing hardware improvements via the use of high-bandwidth piezo actuators and cantilever are only applicable for small-size imaging (less than 30% of the imaging size of regular AFMs [14]), and the existing algorithm improvement based on advanced control techniques may lead to a potential sample deformation and damage due to the lack of control of the tip–sample interaction force [7–8,16]. Therefore, current efforts to high-speed dynamic-mode AFM imaging only led to rather limited success.

The presented AMLM imaging approach can achieve high-speed TM imaging for both large- and small-size imaging while maintaining the image quality and keeping the average tip–sample interaction force at the minimum level for stable cantilever tapping. AMLM imaging is fundamentally different from current efforts to high-speed TM imaging [6,8,15–16] insofar through the introduction of the control of the averaged cantilever deflection (the TM deflection) – in addition to the transitional RMS amplitude feedback control, along with an online iterative feedforward control to track the sample topography. Although this AMLM technique has been proposed recently [1], imaging results of only one polymer sample at large scanning size (50 μm) were obtained and presented. The performance, usability, and particularly, robustness of the AMLM technique for a variety of materials of different topography characteristics and different heterogeneous material properties, and at different imaging sizes have not been yet elucidated. As a result, the AMLM imaging as a highly efficient dynamic-mode AFM imaging technique for wide implementations in practice is still yet to be established. Therefore, it is necessary and crucial to test and evaluate the AMLM technique in various imaging scenarios.

In this work, we present the implementation and evaluation of the AMLM technique by imaging three types of polymer samples different in both topography features and heterogeneous mechanical properties. The three samples, made of polystyrene–low-density polyethylene (PS–LDPE), styrene–butadiene–styrene (SBS) and celgard, respectively, have over two orders of magnitude different lateral feature sizes, ranging from 10 nm (Celgard) to 100 nm (SBS) and up to 2 μm (PS–LDPE), respectively. The elasticity also differs over two orders of magnitude, ranging from 20 MPa (SBS) to 2 GPa (PS). These samples, with largely heterogeneous mechanical properties varying by two orders of magnitude on the same sample, have been widely used as benchmark samples for testing and evaluating AFM imaging. Results of high-speed AMLM imaging (20 Hz and 25 Hz) on these three samples are evaluated and compared to those of TM imaging at much lower speeds (2.5 Hz and 1 Hz) for three largely different imaging sizes (50 μm, 5 μm, and 4 μm). All of the imaging results showed that the AMLM imaging can maintain the same image quality at a scan rate of 25 Hz (with the corresponding probe velocity at 2.5 mm/sec.) as TM imaging at 1 Hz. The tapping amplitude error and the tip–sample interaction force were reduced by over 15% and 27%, respectively, from those of TM imaging at scan rates 10-fold slower (2.5 Hz and 2 Hz). Therefore, through this work, the AMLM clearly stood the test as an efficient high-speed dynamic-mode AFM-imaging technology for practical applications.

## Results and Discussion

**Figure 1:**
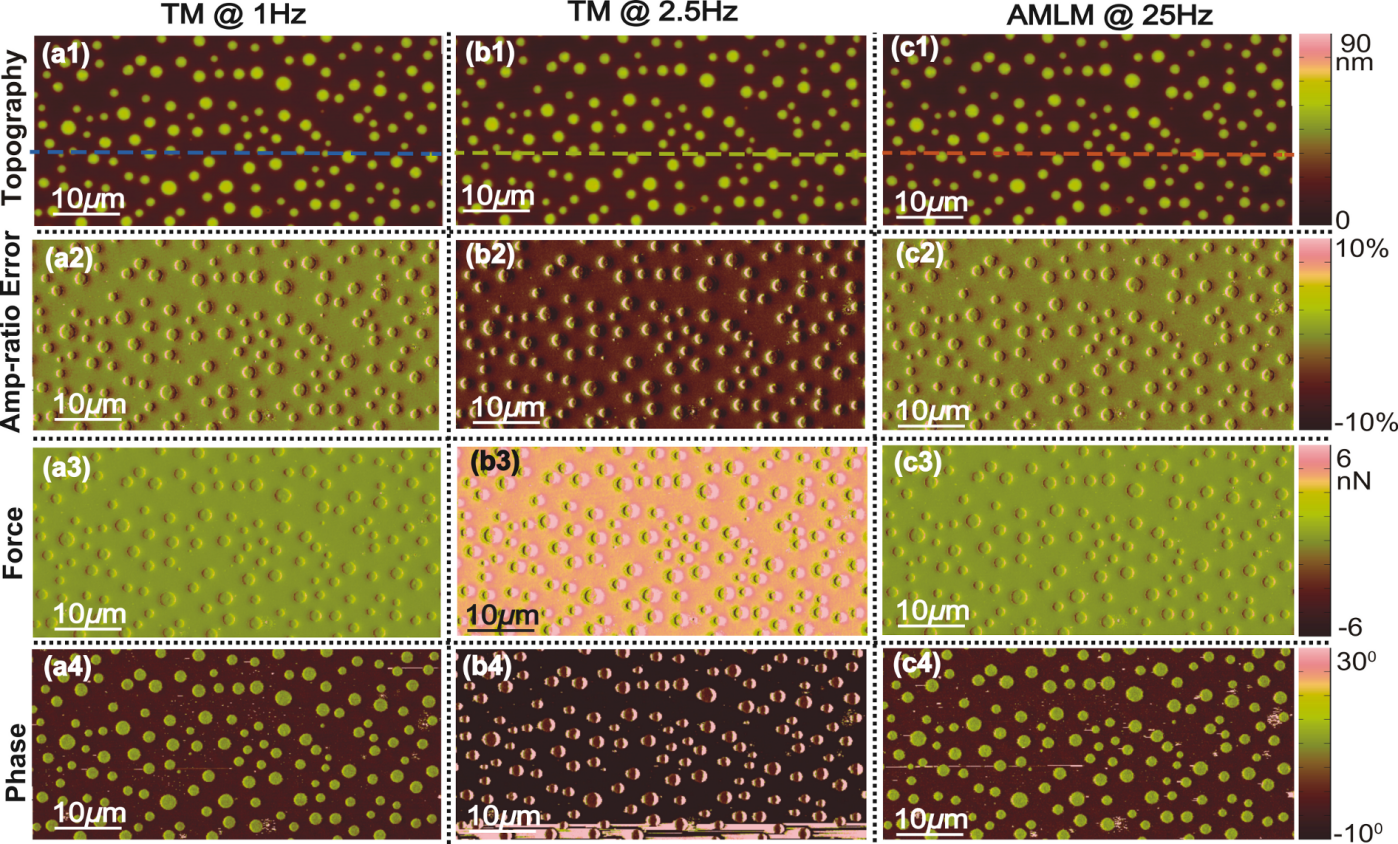
Images of the PS–LDPE sample topography (scan area: 50 μm × 25 μm, scan direction: 50 μm). the corresponding tapping-amplitude-ratio error, the averaged tip–sample interaction force, and the phase contrast obtained using TM imaging at (a1–a4) 1 Hz and (b1–b4) 2.5 Hz, and AMLM imaging at (c1–c4) 25 Hz, respectively.

**Figure 2:**
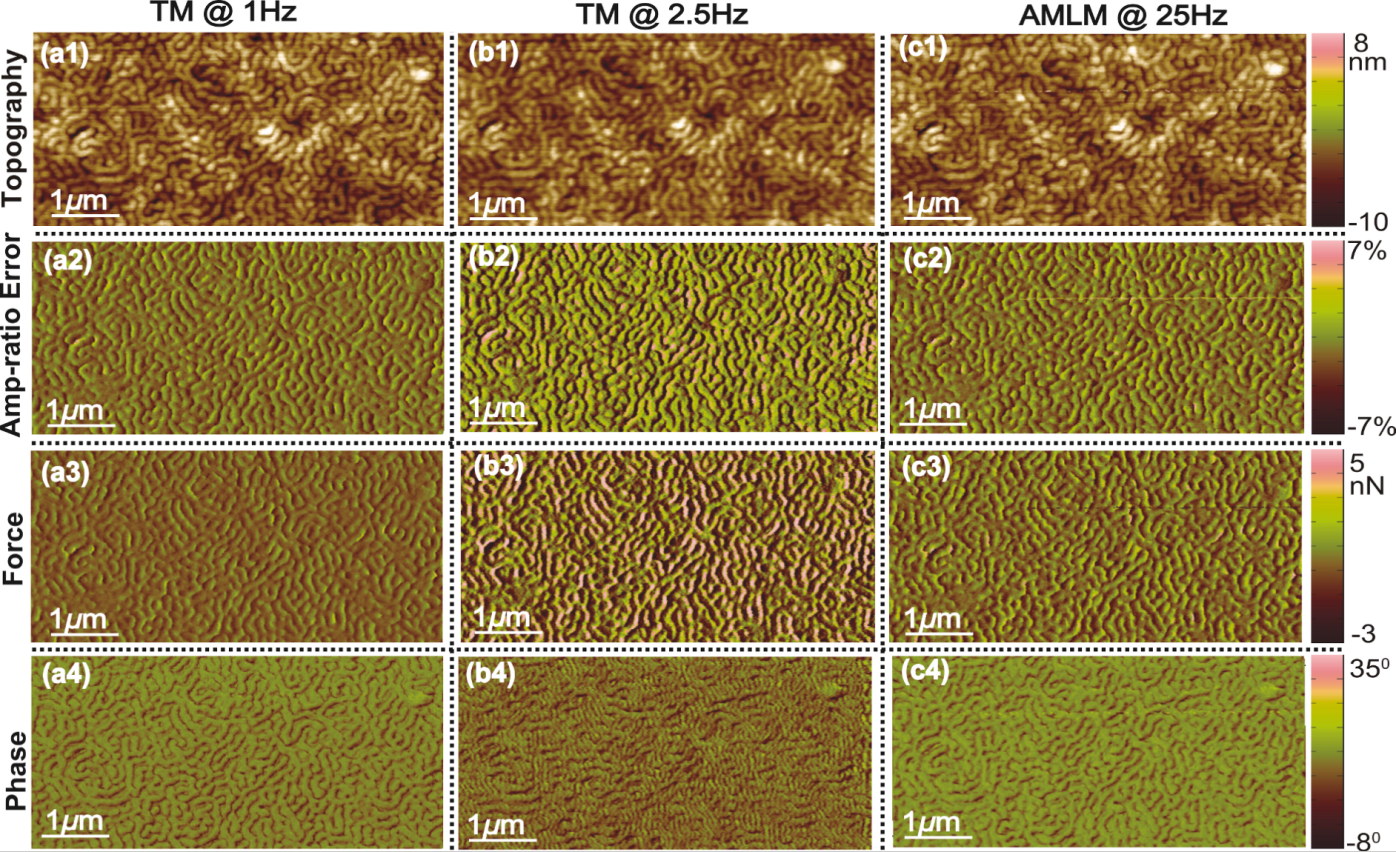
Images of the SBS sample topography (scan area: 5 μm × 2.5 μm, scan direction: 5 μm), the corresponding tapping-amplitude-ratio error, the averaged tip–sample interaction force, and the phase contrast obtained using TM imaging at (a1–a4) 1 Hz and (b1–b4) 2.5 Hz, and AMLM imaging at (c1–c4) 25 Hz, respectively.

**Figure 3:**
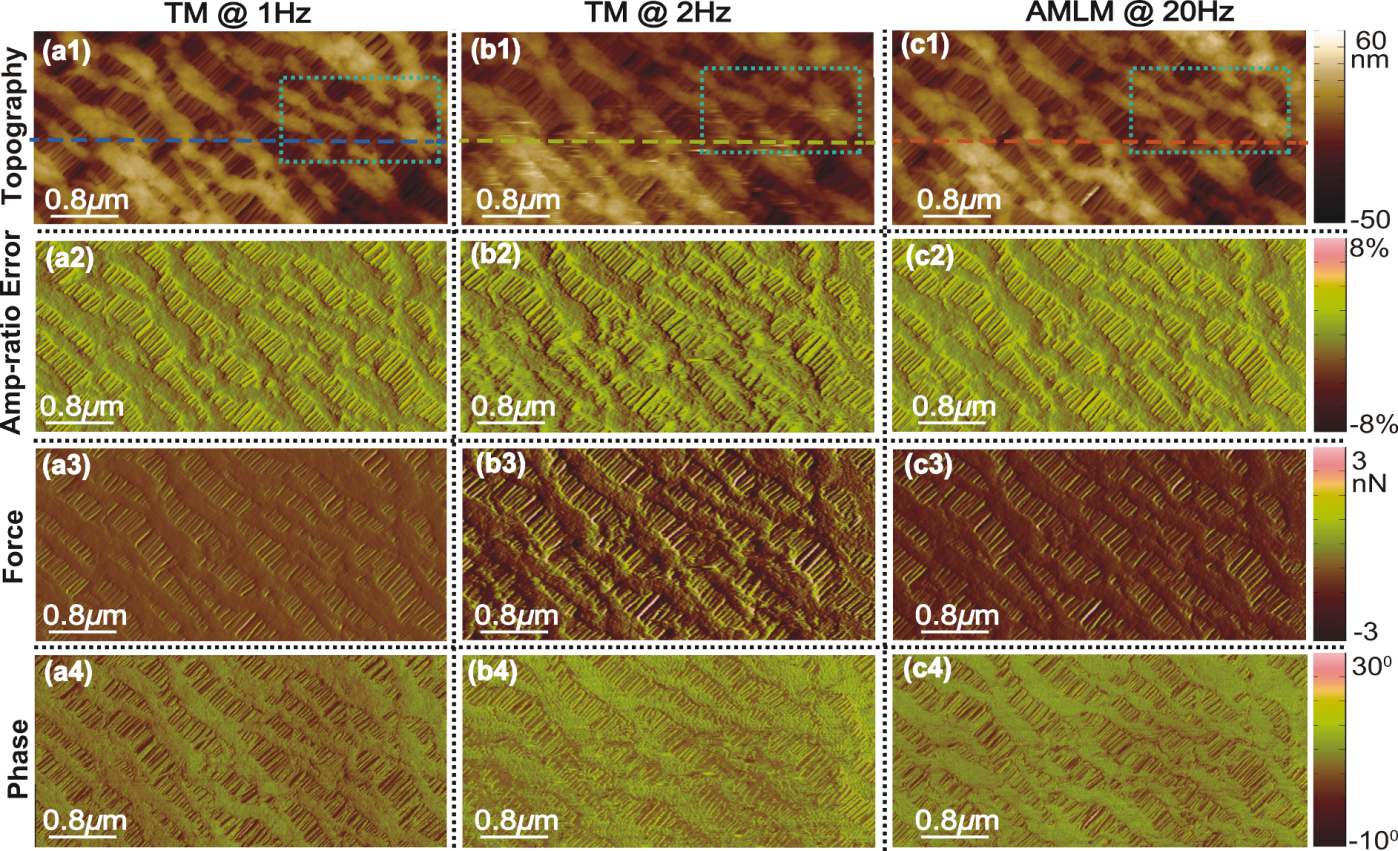
Images of the Celgard sample topography (scan area: 4 μm × 2 μm, scan direction: 4 μm), the corresponding tapping-amplitude-ratio error, the averaged tip–sample interaction force, and the phase contrast obtained using TM imaging at (a1–a4) 1 Hz and (b1 b4) 2 Hz, and AMLM imaging at (c1–c4) 20 Hz, respectively.

**Figure 4:**
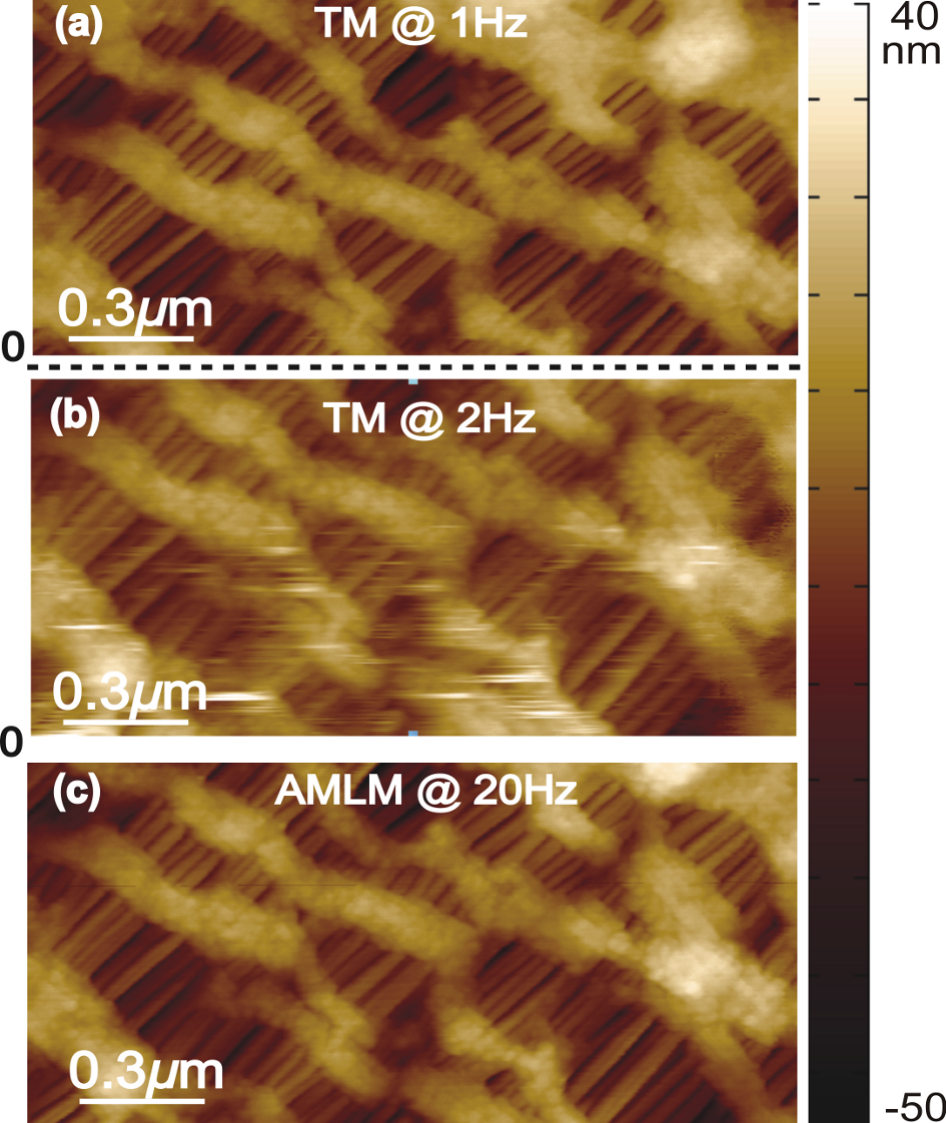
Zoomed-in view of the Celgard sample topography as marked out in Figure 3 obtained using TM imaging at (a) 1 Hz and (b) 2 Hz, and AMLM imaging at (c) 20 Hz.

**Figure 5:**
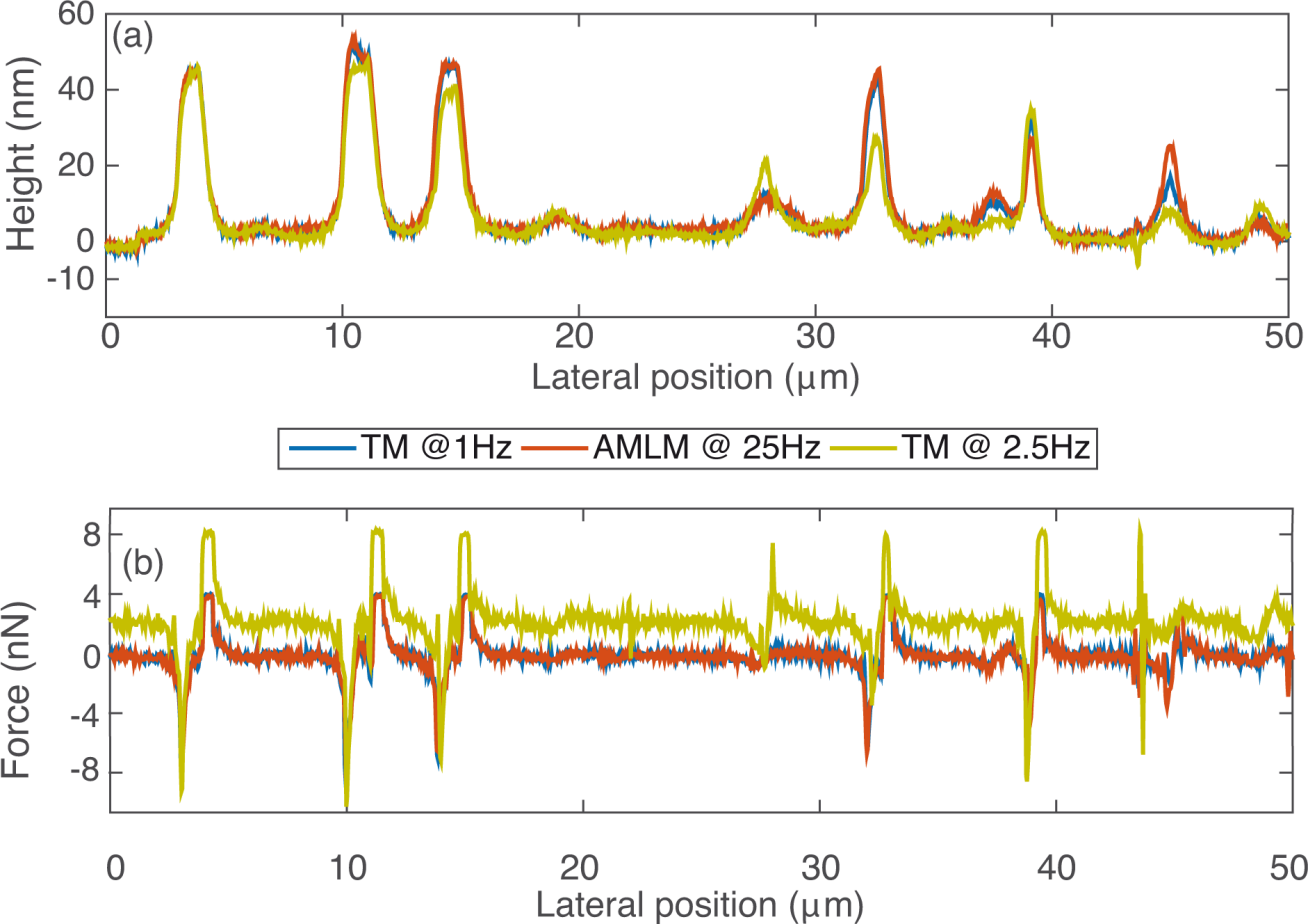
Comparison of (a) the sample topography and (b) the averaged tip–sample interaction force at the cross-section marked out in Figure 1.

**Figure 6:**
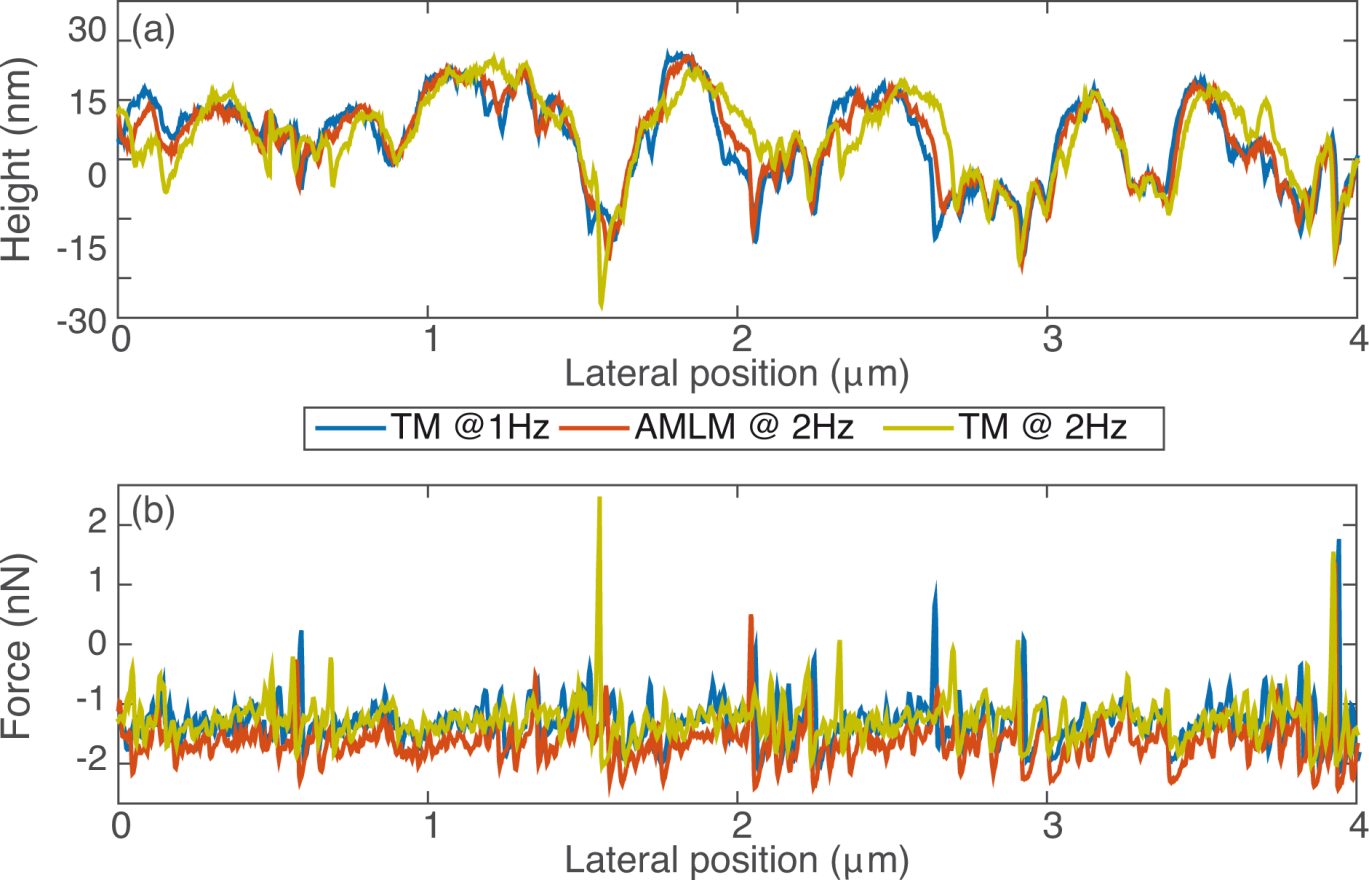
Comparison of (a) the sample topography and (b) the averaged tip–sample interaction force at the cross-section marked out in Figure 3.

AMLM imaging results of the three samples at the scan rates of 25 Hz (for the PS–LDPE and the SBS samples) and 20 Hz (for the Celgard sample) are compared to those obtained using TM imaging at the scan rate of 2.5 Hz (for the PS–LDPE and the SBS samples) and 2 Hz (for the Celgard sample) in Figure 1, Figure 2, and Figure 3, respectively, where the scanning size was 50 μm (PS–LDPE), 5 μm (SBS), and 4 μm (celgard), respectively. The sample topography, tapping-amplitude-ratio error, mean tip–sample interaction force, and phase images are also compared. Moreover, zoomed-in views of the topography images of the Celgard sample are compared in Figure 4, and the cross-section comparison of the sample topography and the mean tip–sample interaction force are compared for the PS–LDPE and the celgard samples in Figure 5 and Figure 6, respectively. The sample topography obtained by 1 Hz TM imaging was used as reference, and the mean tip–sample interaction force was computed using Equation 1.

The imaging results demonstrate the efficacy of AMLM imaging for high-speed dynamic-mode imaging. As shown in Figure 1–Figure 3, the quality of all the AMLM images was clearly improved over that of the TM images at 2.5 Hz and 2 Hz, and was about the same as that of 1 Hz TM imaging. Compared to the 1 Hz TM imaging results as the reference the topography error of the PS–LDPE image obtained using AMLM imaging at 25 Hz was 9%, which is 16% lower than that of the 2.5 Hz TM image. The averaged tip–sample interaction force and the tapping-amplitude-ratio error of AMLM imaging at 25 Hz were, respectively, 43% and 31% lower than those of the TM imaging at 2.5 Hz. Such substantial improvements can also be clearly seen from the cross-section plot in Figure 5. At the cross-section marked in Figure 1, the topography imaging error of the 2.5 Hz TM image, with respect to the 1 Hz TM image, was 18% higher than that of the 25 Hz AMLM image of 8%. As a result, the amplitude of the averaged tip–sample interaction force of the 2.5 Hz TM imaging at that scan-line was 28% higher than that of the 25 Hz AMLM imaging. Note that as the imaging quality needs to be traded-off with the force applied in TM imaging (or in all AFM imaging techniques in general), to achieve the best possible TM images of the PS–LDPE sample at the scan rate of 2.5 Hz in experiments (as shown in Figure 1), the set-point of the amplitude ratio was experimentally tuned and set around 20%, resulting in large probe–sample interaction force. Furthermore, both the phase image obtained using the TM imaging at 1 Hz and that obtained by AMLM imaging at 25 Hz clearly revealed the material property difference between PS and LDPE. In Figure 1, the round dots are made of LDPE, and the rest is PS, with the elasticity of PS and LDPE being 2 GPa and 100 MPa, respectively. However, this information was not discernible in the 2.5 Hz TM phase image (see Figure 1(b4)). Therefore, AMLM imaging yielded high-quality dynamic-mode imaging, and clearly identified the material property difference of heterogeneous polymer samples at high speed and large imaging size.

The performance, usability and robustness of the AMLM technique can be further evaluated through the images of the SBS sample and the Celgard sample with much smaller features than the PS–LDPE sample, at small imaging sizes of 5 μm and 4 μm, respectively. In Figure 2, the “squiggle” feature of the SBS sample was clearly tracked by AMLM imaging at 20 Hz and TM imaging at 1 Hz, but was blurry when using TM imaging at 2.5 Hz. The topography error of the AMLM imaging was 8% with respect to the 1 Hz TM imaging. Moreover, the averaged tip–sample interaction force and the tapping-amplitude-ratio error of the 25 Hz AMLM imaging were 14% and 17%, respectively, different from those of the 1 Hz TM imaging, or equivalently, 42% and 26%, respectively, lower than those of the 2.5 Hz TM imaging. Due to its porous feature at nanometer scale and extremely low tensile strength, Celgard poses a rather daunting challenge for high-speed topography imaging. The experiment results demonstrated that high-quality topography images were achieved at a scan rate of 20 Hz using the AMLM technique. For example, the zoomed-in view of the topography images in Figure 4 showed that the porous feature (at a size around 10 nm) of the Celgard sample was clearly captured in the 20 Hz AMLM image and the 1 Hz TM image, but was seriously distorted in the 2 Hz TM image. The topography error of the AMLM imaging was 11%. Also AMLM imaging was able to maintain the tip–sample interaction force and the tapping-amplitude-ratio error 27% and 11% lower than those of the 2 Hz TM imaging, respectively (both with respect to the corresponding results of the 1 Hz TM imaging). When comparing the sample topography tracking error and the amplitude of the averaged tip–sample interaction force at the cross-section marked out in Figure 3 the 2 Hz TM imaging results were 41% and 27%, respectively, which is 32% and 16% higher than those of the 20 Hz AMLM imaging results, respectively (Figure 6). Therefore, AMLM imaging is also capable of high-speed imaging on polymer samples with much smaller feature size at small scan sizes.

**Figure 7:**
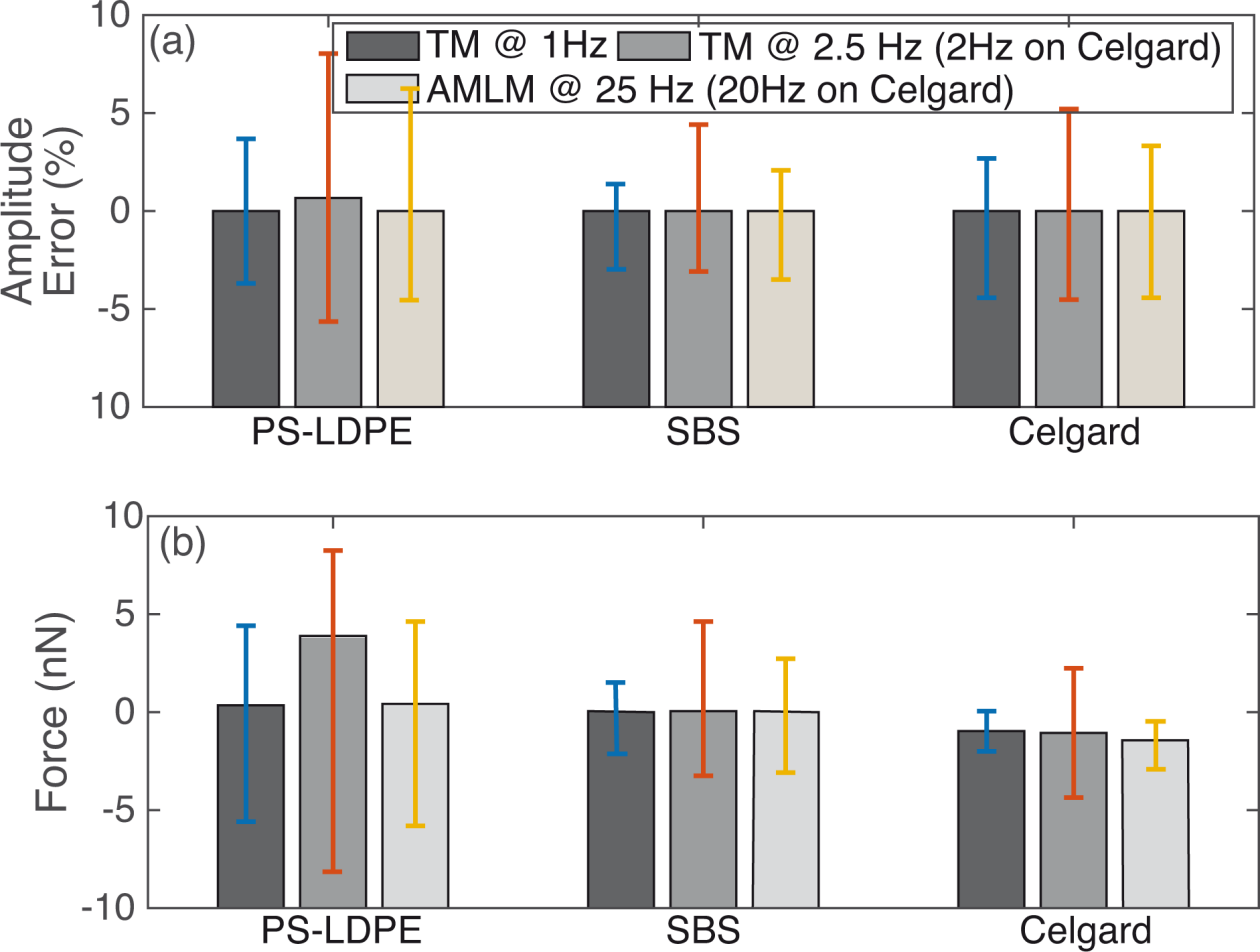
Comparison of (a) the tapping-amplitude-(ratio) error and (b) the averaged tip–sample interaction force over the entire image for 1 Hz TM imaging, 2.5 Hz (2 Hz) TM imaging, and 25 Hz (20 Hz) AMLM imaging of the three tested samples, where the vertical bar denotes the corresponding standard deviation over the entire image.

**Figure 8:**
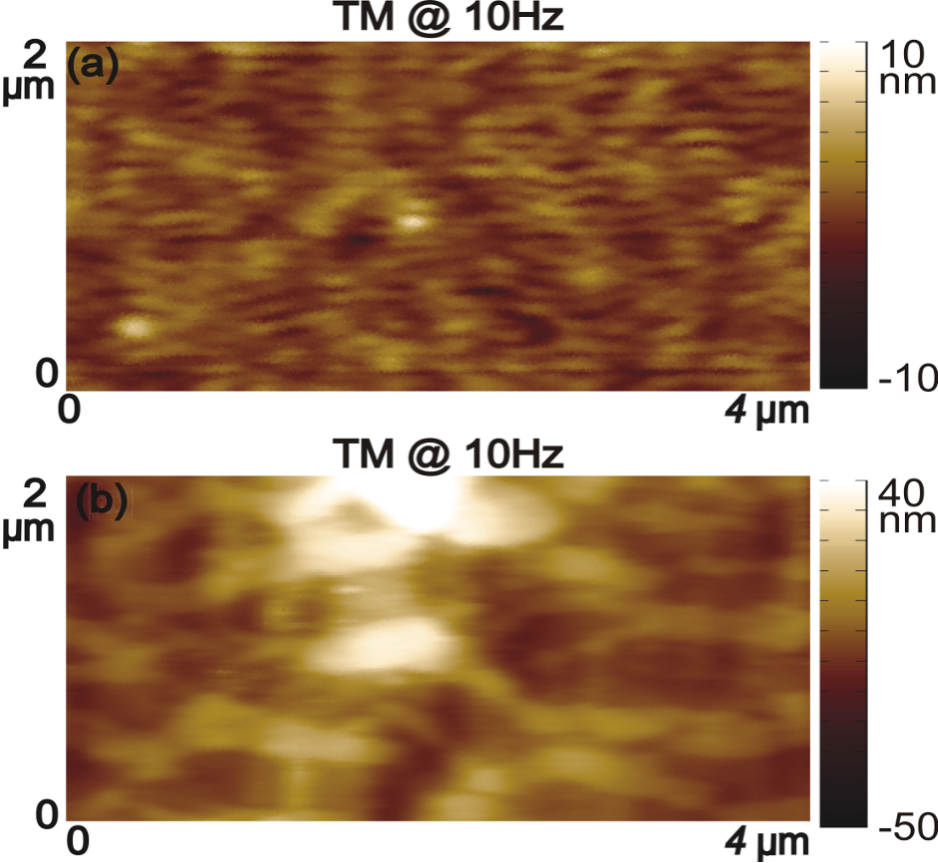
Topography images obtained using the TM imaging at the scan rate of 10 Hz of (a) the SBS sample and (b) the Celgard sample.

Moreover, to quantify the overall performance of the AMLM technique over TM imaging for all the three tested benchmark polymer samples, the tapping-amplitude-ratio error and the mean tip–sample interaction force distributions (the RMS values and the standard deviation) over the entire images are shown in Figure 7. It is clear that by using the AMLM imaging, the tip-sample interaction force was much better controlled than thatwhen using the TM imaging during the imaging of all three tested samples: When using the AMLM imaging, the fluctuation of the tapping amplitude ratio (measured by its RMS value) was only increased slightly by 13%, 17%, and 8% for the PS–LDPE sample, the SBS sample and the Celgard sample, respectively, compared to that of 1 Hz TM imaging, and 31%, 26% and 11% smaller than that of the 2.5 Hz TM imaging, respectively. The mean tip–sample interaction force of AMLM imaging at 25 Hz (and 20 Hz) was slightly increased by 6%, 14%, and 19% from that of 1 Hz TM imaging on the PS–LDPE sample, the SBS sample and the Celgard sample, respectively, and 43%, 42% and 27% lower than that of TM imaging at 2.5 Hz (and 2 Hz), respectively. As a further comparison, the 10 Hz TM topography images of the SBS and the Celgard samples are also shown in Figure 8. It is clear that the 10 Hz TM imaging results were not acceptable. Therefore, the imaging results on the three benchmark samples demonstrated the capability of the AMLM technique in high-quality high-speed imaging on a wide variety of samples with largely different feature sizes and mechanical properties.

## Conclusion

In this work, the performance of the adaptive multiloop-mode (AMLM) imaging technique has been tested and assessed via high-speed imaging of three benchmark polymer samples, namely PS–LDPE, SBS and Celgard. The AMLM imaging results at scan rates of 25 Hz and 20 Hz were compared to those obtained by using TM imaging at scan rates of 2.5 Hz and 2 Hz, respectively. The imaging results demonstrated the efficacy of the AMLM imaging approach and its superiority over commercial TM imaging in both small- and large-size imaging. AMLM imaging maintained the imaging quality at scan rates of 25 Hz and 20 Hz as that of the TM imaging at 1 Hz, and reduced the tapping-amplitude-ratio error and the mean tip–sample interaction force by over 15% and 27%, respectively, from those of TM imaging at 10-fold slower scan rates (2.5 Hz and 2 Hz). Therefore, the experimental results clearly demonstrated the AMLM as a highly efficient practical technique for high-speed dynamic-mode imaging of a wide variety of heterogeneous samples.

## Experimental

### Adaptive multiloop-mode imaging

The AMLM aims to achieve high-speed dynamic-mode imaging by precisely tracking the sample topography, while minimizing the mean tip–sample interaction force per vibration period, 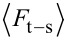.

**Figure 9:**
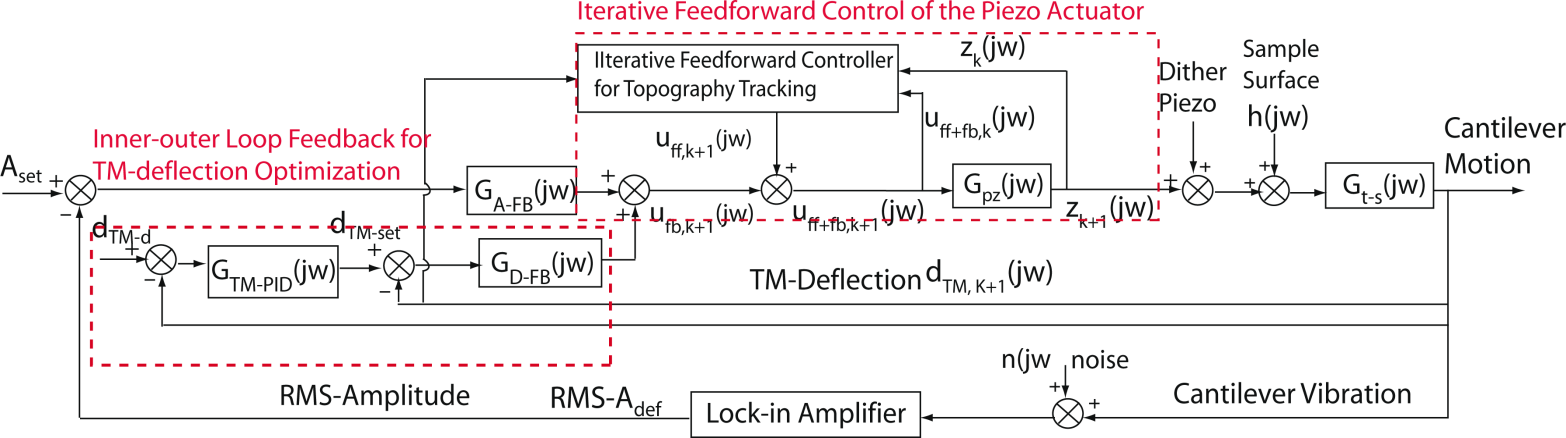
Schematic block diagram of the proposed AMLM imaging. This figure is an adapted version from [1].

The key to the optimization of the mean tip–sample interaction force is to accurately track the sample topography by the AFM *z*-axis piezo. AMLM imaging introduces a feedback control of inner–outer loop structure to regulate the mean cantilever deflection per vibration period (called the TM-deflection). Thus the averaged (vertical) position of the cantilever in each tapping period is kept closely around the desired value (see Figure 9) [1]. The outer loop adjusts the TM-deflection set-point in real time, while the inner loop tracks the adjusted TM-deflection set point. The following extended gradient-based approach is employed to adjust the TM-deflection set-point, *d_TM−set_*(·),

[2]
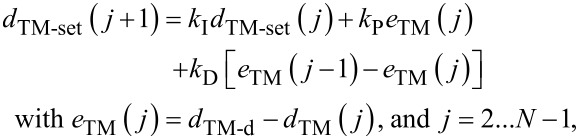


where *N* is the total number of sampling periods per image, *d*_TM_(*j*) is the TM-deflection of the current sampling point, and *k*_P_, *k*_I_, and *k*_D_ are the proportional (P), integral (I), and derivative (D) parameters, respectively. The desired TM-deflection, *d*_TM−d_, is determined by the ratio of the chosen tapping-amplitude set-point to the free amplitude, *A*_set_/*A*_free_, based on a priori measured relation between *d*_TM−d_ and (*A*_def_/*A*_free_) [1].

An online data-driven iterative feedforward controller is integrated to the RMS-*z*-feedback loop to enhance the sample topography tracking (Figure 9). Particularly, the feedforward control input is obtained by implementing the following high-order modeling-free difference-inversion-based iterative-control (HOMDIIC) algorithm [1,17] online,

[3]
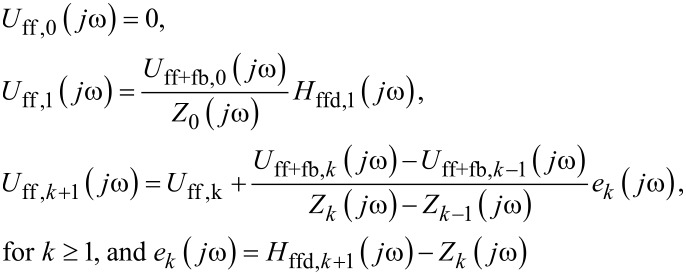


where “*jω*” denotes the Fourier transform of the corresponding signal, and *U*_ff+fb,_*_k_*(·) and *Z_k_*(·) are the total control input (feedback+feedforward) applied to the *z*-piezo actuator (i.e., *U*_ff+fb,_*_k_*(*jω*) = *U*_ff,_*_k_*(*jω*) + *U*_fb,_*_k_*(*jω*), see Figure 9), and the *z*-piezo displacement measured on the *k*-th scan line, respectively, and *H*_ffd,_*_k_*_+1_(·) denotes the desired trajectory that the *z*-piezo needs to track at the (*k*+1)-th scanline. Note that the ratio in the above control law, (*U*_ff+fb,_*_k_*(*jω*) − *U*_ff+fb,_*_k_*_−1_(*jω*))/(*Z_k_*(*jω*) − *Z_k_*_−1_(*jω*)), essentially equals the inverse of the frequency response of the *z*-piezo actuator, and is updated line-by-line iteratively throughout the whole imaging process.

Furthermore, the design of *H*_ffd,_*_k_*(·) in Equation 3 takes into account both the sample topography of the current scan line and the TM-deflection tracking error, *d*_TM,_*_k_*(*j*) − *d*_TM−d_ [1] as follows

[4]
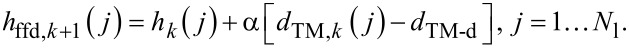


Here, *N*_l_ and α are the total sampling points per scan-line and the correction factor, respectively. Details of implementation the iterative scheme in AMLM can be found in [1].

As the TM-deflection responds much faster than the RMS tapping-amplitude to the sample topography variation, so the inner-outer loop feedback control of the TM-deflection is faster than that of the RMS tapping amplitude. Moreover, the TM-deflection feedback loop facilitates the regulation of the tapping amplitude, and further minimizes the averaged probe–sample interaction force (by regulating the TM-deflection closely around the desired value). The feedforward controller further reduces the fluctuation of the tapping amplitude upon sudden sample topography variation.

To ensure the accuracy of sample topography quantification, AMLM imaging takes both the AFM *z*-piezo displacement and the TM-deflection into account [1] in quantification of *h_k_*(*j*). In particular, the sample surface height at any given point can be quantified with respect to a fixed reference point with *h*_0_ = 0 and the corresponding *d*_TM,0_ = *d*_TM−d_ [1],

[5]
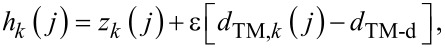


where ε is the contact constant that depends on the tip–sample interaction regime: ε = −1 when the tip–sample interaction is dominated by the long-range attractive force. ε = 1 when a repulsive tip–sample interaction force appears. For 
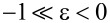
 the interaction force vanishes (i.e.,the tapping amplitude is close to the free vibration amplitude).

Finally, the mean tip–sample interaction force (per tapping period *T*), 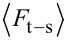, can be quantified as [1,10–11],

[1]
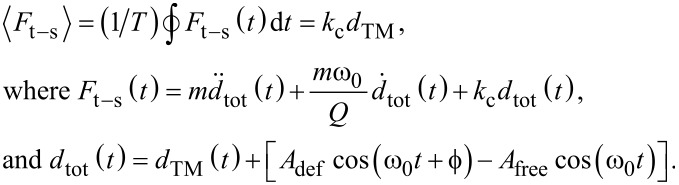


where *k*_c_ and *m* are the spring constant and the mass of the cantilever, respectively, *Q* is the quality factor of the cantilever, *d*_tot_(*t*), *A*_def_ and *A*_free_ are the total deflection, the tapping amplitude, and the free-vibration amplitude of the cantilever, respectively, and ω_0_ is the tapping frequency.

### Implementation of AMLM imaging

To test and evaluate the AMLM imaging technique, three benchmark samples (a PS–LDPE sample, an SBS sample, and a Celgard sample) were imaged using the AMLM approach on an AFM system (Dimension Icon, Bruker Nano Inc.) along with a tapping-mode cantilever (MPP-21120-10, Bruker Inc., nominal spring constant: 3 N/m, resonant frequency: 75 kHz). Both the conventional TM imaging and AMLM imaging were designed and implemented in a Matlab xPC-target (R2010a) system, and applied to the AFM imaging through a computer-based data acquisition system (NI-6259, National Instrument, sampling frequency: 20 kHz). The AFM system was modified to allow for direct acquisition of all the required sensor signals and direct application of the driven signals to the AFM piezo actuators. Before imaging, the HODMIIC technique (Equation 3) was employed to achieve precise tracking in the lateral *x*–*y* axes scanning [17] (the RMS tracking error was maintained below 1%). Then the converged inputs for *x*- and *y*-piezo actuators were applied during the imaging. Tapping at 30% of the free vibration of 120 nm was chosen so that the tapping motion of the cantilever was maintained and the corresponding mean TM-deflection was closely around zero, thereby, minimizing the corresponding mean vertical probe–sample interaction force. To make a fair comparison between AMLM and TM, the amplitude set-point in all TM imaging was not fixed and tuned in order to get the best possible images.
